# Generational Perspectives on Technology's Role in Mental Health Care: A Survey of Adults With Lived Mental Health Experience

**DOI:** 10.3389/fdgth.2022.840169

**Published:** 2022-02-10

**Authors:** Molly Woerner, Nichole Sams, Cristian Rivera Nales, Tara Gorstein, Morgan Johnson, Brittany A. Mosser, Patricia A. Areán

**Affiliations:** ^1^Conducting Research to Enhance Assessment and Treatment Through Innovation in Mental Health (CREATIV) Lab, Department of Psychiatry and Behavioral Sciences, University of Washington, Seattle, WA, United States; ^2^Advanced Laboratories for Accelerating the Reach and Impact of Treatments for Youth and Adults With Mental Illness (ALACRITY Center), Department of Psychiatry and Behavioral Sciences, University of Washington, Seattle, WA, United States; ^3^Adolescent Adversity and Depression Intervention (AADI) Lab, Department of Psychiatry and Behavioral Sciences, University of Washington, Seattle, WA, United States

**Keywords:** older adults, digital mental health, lived experience, technology, mental health

## Abstract

**Introduction:**

Personal technology (e.g., smartphones, wearable health devices) has been leveraged extensively for mental health purposes, with upwards of 20,000 mobile applications on the market today and has been considered an important implementation strategy to overcome barriers many people face in accessing mental health care. The main question yet to be addressed is the role consumers feel technology should play in their care. One underserved demographic often ignored in this discussion are people over the age of 60. The population of adults 60 and older is predicted to double by 2,050 signaling a need to address how older adults view technology for their mental health care.

**Objective:**

The objective of this study is to better understand why digital mental health tools are not as broadly adopted as predicted, what role people with lived mental health experience feel technology should play in their care and how those results compare across age groups.

**Method:**

In a mixed-methods approach, we analyzed results from a one-time cross-sectional survey that included 998 adults aged 18–83 with lived experience of mental health concerns recruited from Prolific, an online research platform. We surveyed participant's use of technology including their perspectives on using technology in conjunction with their mental health care. We asked participants about their previous use of digital mental health tools, their treatment preferences for mental health care, and the role technology should play in their mental health care.

**Results:**

Across all age groups, respondents had favorable views of using digital mental health for managing mental health care. However, older adults rated their acceptability of digital mental health tools lower than middle-aged and younger adults. When asked what role technology should play in mental health care in an open-ended response, most participants responded that technology should play a complementary role in mental health care (723/954, 75.8%).

**Conclusion:**

Digital mental health is seen as a valuable care management tool across all age groups, but preferences for its role in care remain largely administrative and supportive. Future development of digital mental health should reflect these preferences.

## Introduction

There is no question that access to mental health services in the US is difficult; 58% of people with mental illness never receive treatment (1). Decades of research has shown that poor access is due to several factors, from the stigma people experience when they ask for help to the fact that there are severe mental health provider shortage areas particularly in rural areas or urban poverty areas (2). Even in high provider density areas, older adults remain sorely underserved because too few mental health providers are credentialed in geriatric mental health (3), and few providers accept Medicaid or Medicare (4). For the US, this shortage will result in significant societal costs, particularly in the case of older adults; it is projected by the year 2040, the number of individuals 85 and older will increase by 129% (5–7). Approximately 20% of older adults will experience a mental health issue, with the most common diagnoses being Major Depressive Disorder, Bipolar Disorder and Schizophrenia (8). Additionally, 17.9% of all suicide deaths are of older adults (9). Access to mental health care in this demographic is important to address.

Since the advent of COVID-19, attention has turned to the potential opportunity digital mental health (apps, tele-health, and message-based care) has in overcoming access problems and mental health disparities. According to a recent Banbury Report on digital mental health (10) such technologies may address the mental health needs of US citizens; these tools are evidence-based and prolific, with over 20,000 apps and 40 companies offering these services (11). Further, digital mental health is cost-effective, scalable, lowers the cost of hospitalizations, lowers burden on providers, increases access to care in rural settings where broadband is available, results in lower wait times, and can personalize treatment (12–14).

Although digital mental health tools are an effective and efficient mental health service delivery method, very few people with mental health needs use these tools. Even during COVID-19, only 16% of essential workers and unemployed individuals in need of mental health care ever used a digital mental health tool, with 86% of those with mental health distress during COVID-19 indicating that they did not believe such tools would help (15). Other studies have found that large proportions of the general population have similar quality concerns in addition to security and safety concerns about digital mental health care (16). Older adults are less likely than younger adults to use digital mental health, although this trend may be changing due the increase in general technology use by older adults during COVID-19 (17). For older adults, utilization challenges may be driven by challenges they experience when using mobile technology, such as trouble understanding application interfaces, dexterity issues, preference for human interaction, and cognitive issues (18–20). Taken together these studies suggest that underutilization of digital mental health tools may be in large part due to its acceptability as a mental health care option, and, for older adults, usability challenges and preference for care delivery.

To date the field has not included representative user's perspectives on the role technology should play in mental health recovery, and relative preferences among digital options and in-person care, particularly among older populations. According to Human Centered Design Theory (21, 22), the needs and values of intended audiences or users, in this case people with lived experience in mental health, must be central to determining the role technology plays in solving a problem, its design, and its purpose. The purpose of this study is to better understand why digital mental health tools are not as broadly adopted as predicted, what role people with lived mental health experience feel technology should play in their care and in particular the accessibility challenges of digital mental health by age group. Our specific aims are: (1) to ask people with lived experience with mental health concerns the role technology should play in their care, (2) their preferences among different digital mental health care options, and (3) compare responses by age group.

## Methods

### Study Design

This was a cross-sectional, remote survey of digital mental health tool use.

### Participants and Procedures

One thousand thirty-two research participants were recruited using Prolific (www.prolific.co), an online research platform that allows researchers to screen, recruit, enroll, and pay participants. Participants in this study were 18 years or older, English speaking, living in the United States.

All participants endorsed having experienced a mental health condition or treatment on a pre-screening survey. The questions of this pre-screening survey included (1) “Have you ever received help in the past for stress or mental health issues? The help may have been psychotherapy, counseling, and/or medication for depression, anxiety, stress management or other mental health issue,” (2) “Are you currently receiving help (psychotherapy, counseling, medication) for stress or mental health issues?,” and (3) “Have you ever experienced a mental health condition, such as depression, anxiety, or psychosis?” Participants were excluded if they did not endorse “yes” to at least one of these three questions.

After recruited participants completed the pre-screening survey using Research Electronic Data Capture (REDCap) and met eligibility criteria, they were given a link to complete the full survey on REDCap (23, 24). Recruitment was stratified by US census racial categories to include a racially representative sample. We oversampled under-represented minority populations in an attempt to obtain a sample consistent with US Census representation. We also oversampled older adults to ensure a large enough older adult sample to accurately report on age differences. Participants were paid $0.50 for completing the pre-screening survey and $5.00 for completing the full survey.

### Ethics Statement

The study was approved by the University of Washington's Institutional Review Board (STUDY00014041; FWA #00006878).

### Data Collection

Participants completed the survey on REDCap. The survey took an average 20 min to complete and included sections on demographics, health and wellbeing, use of technology, use of apps, social media and the internet, treatment preferences, and a “Build Your Own Mental Health Experience” section. See Supplementary Material for full survey questions. Although participants provided answers to several questions about comfort with technology, use of technology for physical health and preferences within mental health tools, we report here only on data concerning current use of mental health technology, preferences for different types of digital mental health technology, and participant's perspectives on the role that technology should play in mental health care.

### Protection Against Malicious Actors

In interest of data quality, several procedures were enacted to prevent “bad actors” (individuals or entities who participate in bad faith to accumulate monetary incentives) from participating in the study and joining the final study sample. Prolific research platform enacts measures to vet participants such as verification of email address, phone number (ensuring it correlates with their country of residence), photo identification, and PayPal address (25). Technical measures include restricting signups based on IP address and ISP, requiring a unique non-VOIP phone number, and analysis of data to review unusual patterns (26). A PayPal or Circle account is required to be paid and both have procedures to prevent duplicate accounts. We included two attention checks in our survey that required participants to read questions fully and answer them both accurately to be included in the final sample. By including open-ended questions in our study, we were able to screen any non-coherent answers and address possible malicious actors like bots (27). Both specific platform quality checks and our internal quality checks assure our final sample is comprised of quality “good actors.”

### Demographics

Participants were asked to provide their age, zip code, description of community size, employment level, number of people living in home, if someone identifies as an underrepresented person, US Census racial categories, ethnicity, gender identity, and financial comfort. All participants were stratified in one of three age ranges: (1) younger adults (YA: 18–34 years, mean = 24.6 years), (2) middle aged adults (MA: 35–59 years, mean = 50.5 years), and (3) older adults (OA: 60+ years, mean = 66.0 years). Although young, middle, and older adulthood is not clearly defined by age we determined age ranges based on common developmental and social periods of adulthood including the transitionary periods of family roles, careers, and changing health (28). Younger adulthood comprises of pursing new careers, social relationships, and identity formation (29). Middle age presents new life circumstances such as new family demands, career seniority, and new health challenges (30). The older adult population is characterized as a period with changes in cognition, physical health, fewer work and family responsibilities, and increased focus on meaningful aging and experiences toward the end of life (31). We asked participants to describe their current health and general wellbeing to characterize the sample on current physical and emotional functioning. To assess current levels of emotional distress, we asked participants to complete the 9-item Patient Health Questionnaire (PHQ-9) (32) which asks about the frequency and severity of depressive symptoms over the previous 2 weeks, and the 7-item General Anxiety Disorder (GAD-7) (33) which asks about the frequency and severity of generalized anxiety symptoms over the previous 2 weeks.

### Survey Questions

#### Use of Technology for Mental Health

We used questions from a previous survey of preferences for digital mental health among COVID-19 essential workers and those unemployed during the pandemic (15), modified slightly for this study. The survey asked participants if they have ever used technology to manage mental health problems and perspectives on different types of digital mental health tools, specifically apps, message-based care and telehealth. The survey also asked participant preferences for these different mental health technologies. See Supplementary Material for full survey.

#### Role of Technology in Mental Health

Participants were asked a single open-ended question about their thoughts about the role of technology in mental health care. Specifically, they were asked “In your own words, what role should technology serve in mental health care?”

### Statistical Analysis Plan

The original sample included 1,032 participants. Data cleaning removed 34 individuals from the original sample because they either (1) had a duplicate Prolific ID (*n* = 2), (2) did not move past consent (*n* = 1), or (3) did not pass both attention checks (*n* = 31). The final sample included 998 participants.

For each aim, we analyzed differences across age groups. Age was categorized into three groups (1) younger adults (YA: 18–34 years), (2) middle aged adults (MA: 35–59 years), and (3) older adults (OA: 60+ years). To examine differences between age groups, we used chi-square tests for categorical variables, Kruskal–Wallis tests for ordinal variables, and one-way analysis of variance (ANOVA) for continuous variables. When statistically significant differences across groups were found for variables with multiple discrete categories, we conducted *post-hoc* probes using standardized residuals to identify which categories were responsible for the significant difference.

To control the prevalence of false positives due to multiple testing, we applied the Benjamini-Hochberg (B-H) procedure with the false discovery rate set to 10% to 45 statistical tests (34, 35) All statistical analyses were performed with SAS version 9.4.

### Qualitative Analysis

Responses to the question, “In your own words, what role should technology play in your mental health care?” was coded using thematic, content analysis (36). Codes and their definitions were developed by two independent reviewers (M.W. and N.S.) who identified emergent themes in the data. Themes were then codified and confirmed by both reviewers. In the event reviewers did not agree on themes, a third reviewer (P.A.A.) was available to resolve the discrepancy. However, in this project, no third review was needed. We tallied the frequency that a theme was endorsed by different participants to rank themes by prominence in the data. Representative quotes were selected for each theme and are reported in the results section.

## Results

### Sample Characteristics

Table 1 shows frequencies, means, and standard deviations of participant demographics and clinical characteristics. Most of the sample identified as female (651/995, 65.4%), were employed (610/981, 62.2%) and did not self-identify as an underrepresented population based on skin color, heritage, socio-economic status, gender-identity, sexual orientation, or other identified characteristics (654/989, 66.1%). The sample was predominately white (750/994, 75.5%) followed by Black or African American (74/994, 7.4%), multi-racial (69/994, 6.9%), and Hispanic/Latinx (55/994, 5.5%).

**Table 1:** Demographics stratified by age (categorized).

	**Age categorized**			
	**Younger adults (18–34 years, mean = 24.6) (*n* = 290)**	**Middle aged adults (35–59 years, mean = 50.5) (*n* = 406)**	**Older adults (60 + years, mean = 66.0) (*n* = 302)**	**Total (*N* = 998)**
**Employment**				
Unemployed	87 (30.5%)	75 (18.8%)	141 (47.3%)	303 (30.9%)
Unpaid work at home (e.g., primary unpaid caregiver of family member)	10 (3.5%)	33 (8.3%)	6 (2.0%)	49 (5.0%)
Unpaid work out of the home (e.g., volunteerism)	6 (2.1%)	3 (0.8%)	10 (3.4%)	19 (1.9%)
Part time paid work outside the house	63 (22.1%)	29 (7.3%)	21 (7.0%)	113 (11.5%)
Part time paid work at home	16 (5.6%)	42 (10.6%)	50 (16.8%)	108 (11.0%)
Full time paid work outside the house	80 (28.1%)	139 (34.9%)	40 (13.4%)	259 (26.4%)
Full time paid work at home	23 (8.1%)	77 (19.3%)	30 (10.1%)	130 (13.3%)
Missing	5	8	4	17
**Household members**				
1	69 (27.3%)	116 (34.8%)	152 (69.4%)	337 (41.9%)
2	77 (30.4%)	92 (27.6%)	49 (22.4%)	218 (27.1%)
3	66 (26.1%)	69 (20.7%)	12 (5.5%)	147 (18.3%)
4	24 (9.5%)	40 (12.0%)	5 (2.3%)	69 (8.6%)
5+	17 (6.7%)	16 (4.8%)	1 (0.5%)	34 (4.2%)
Missing	37	73	83	193
**Identify as an underrepresented population based on skin color, heritage, socio-economic status, gender-identity, sexual orientation, or other aspects of your identity**.				
Yes	129 (44.6%)	111 (27.7%)	38 (12.7%)	278 (28.1%)
No	140 (48.4%)	261 (65.1%)	253 (84.6%)	654 (66.1%)
Not sure	20 (6.9%)	29 (7.2%)	8 (2.7%)	57 (5.8%)
Missing	1	5	3	9
**Race**				
Multi-racial	29 (10.0%)	33 (8.1%)	7 (2.3%)	69 (6.9%)
American Indian or Alaska native or indigenous	0 (0.0%)	4 (1.0%)	1 (0.3%)	5 (0.5%)
Asian	16 (5.5%)	20 (4.9%)	1 (0.3%)	37 (3.7%)
Black or African American	28 (9.7%)	38 (9.4%)	8 (2.7%)	74 (7.4%)
Hispanic/Latinx	30 (10.4%)	22 (5.4%)	3 (1.0%)	55 (5.5%)
Middle Eastern or North African	3 (1.0%)	1 (0.2%)	0 (0.0%)	4 (0.4%)
White	183 (63.3%)	287 (70.9%)	280 (93.3%)	750 (75.5%)
Missing	1	1	2	4
**Ethnicity**				
Hispanic/Latinx	47 (16.6%)	52 (12.9%)	4 (1.3%)	103 (10.4%)
Non-Hispanic/Latinx	236 (83.4%)	351 (87.1%)	296 (98.7%)	883 (89.6%)
Missing	7	3	2	12
**Gender identity**				
Female	203 (70.0%)	256 (63.4%)	192 (63.8%)	651 (65.4%)
Male	66 (22.8%)	141 (34.9%)	108 (35.9%)	315 (31.7%)
Transgender, Non-binary, or Gender- nonconforming	21 (7.2%)	7 (1.7%)	1 (0.3%)	29 (2.9%)
Missing	0	2	1	3
**Financial stability**, *n* (%)				
Can't make ends meet	35 (12.3%)	62 (15.5%)	35 (11.7%)	132 (13.4%)
Have just enough to get by	162 (56.8%)	164 (41.0%)	137 (45.7%)	463 (47.0%)
Are comfortable	88 (30.9%)	174 (43.5%)	128 (42.7%)	390 (39.6%)
Missing	5	6	2	13
**Home location**, *n* (%)				
Large city	92 (31.9%)	92 (22.7%)	50 (16.6%)	234 (23.5%)
Suburb near a large city	96 (33.3%)	150 (36.9%)	106 (35.1%)	352 (35.3%)
Small city or town	72 (25.0%)	109 (26.8%)	86 (28.5%)	267 (26.8%)
Rural area	28 (9.7%)	55 (13.5%)	60 (19.9%)	143 (14.4%)
Missing	2	0	0	2
**Clinical characteristics**				
**PHQ-9 total score**				
*N*	290	406	302	998
Mean (SD)	10.1 (5.5)	7.5 (5.3)	5.6 (4.2)	7.7 (5.3)
**GAD-7 total score**				
*N*	290	406	302	998
Mean (SD)	9.6 (5.9)	6.2 (5.6)	4.2 (4.5)	6.6 (5.8)

Older adults were more likely to have received help in the past for stress or mental health issues ($\chi_{2}^{2}$ = 11.3, *p* = 0.0035) while no differences emerged for current mental health treatment use or past lived experience across groups.

Overall, the sample's average score on the PHQ-9 and GAD-7 indicated mild levels of depression (mean 7.7, SD 5.3) and anxiety (mean 6.6, SD 5.8) severity. Older adults demonstrated the lowest levels of depression (mean 5.6, SD 4.2) and anxiety (mean 4.2, SD 4.5) severity.

### Survey Results

#### App, Message Based Care, and Telehealth Usage

Table 2 presents app, message-based care, and telehealth usage across age groups.

**Table 2:** App, message based care, and telehealth usage stratified by age categorized.

	**Age Categorized**				
	**Younger Adults (18–34 years) (*n* = 290)**	**Middle Aged Adults (35–59 years) (*n* = 406)**	**Older Adults (60+ years) (*n* = 302)**	**Total (*N* = 998)**	***P*-value**
**App usage**					
**Have you considered using an app for your mental health?**					**<0.01** ^**1**^
No	131 (46.0%)	274 (67.8%)	249 (83.3%)	654 (66.2%)	
Yes	154 (54.0%)	130 (32.2%)	50 (16.7%)	334 (33.8%)	
Missing	5	2	3	10	
**Have you downloaded a mental health app?** ^a^					0.27^1^
No	94 (61.0%)	90 (69.2%)	35 (70.0%)	219 (65.6%)	
Yes	60 (39.0%)	40 (30.8%)	15 (30.0%)	115 (34.4%)	
**Message based care usage**					
**Message-based care can play an important role in managing my mental health**.					**<0.01** ^**2**^
*N*	288	405	301	994	
Mean (SD)	4.3 (1.2)	4.2 (1.2)	4.0 (1.3)	4.2 (1.2)	
Median	5.0	4.0	4.0	4.0	
**I would use message-based care to manage my mental health**.					**<0.01** ^**2**^
*N*	284	405	301	990	
Mean (SD)	4.2 (1.3)	4.1 (1.3)	3.8 (1.4)	4.0 (1.4)	
Median	4.5	4.0	4.0	4.0	
**Message-based care can be an effective intervention for managing mental health conditions**.					**<0.01** ^**2**^
*N*	284	403	298	985	
Mean (SD)	4.4 (1.1)	4.2 (1.2)	4.0 (1.2)	4.2 (1.2)	
Median	4.0	4.0	4.0	4.0	
**If science found that message-based care is effective for managing mental health conditions, I would use it**.					**<0.01** ^**2**^
*N*	284	404	299	987	
Mean (SD)	4.6 (1.2)	4.4 (1.3)	4.1 (1.3)	4.4 (1.3)	
Median	5.0	5.0	4.0	5.0	
**I believe using only message-based care, without video-conferencing, can be effective in managing my mental health**.					0.79^2^
*N*	282	400	299	981	
Mean (SD)	3.3 (1.3)	3.3 (1.4)	3.3 (1.4)	3.3 (1.4)	
Median	3.0	4.0	3.0	3.0	
**I would prefer to use message-based care (without video-conferencing) rather than video-conferencing to manage my mental health**.					0.46^2^
*N*	286	402	298	986	
Mean (SD)	3.2 (1.6)	3.3 (1.5)	3.2 (1.6)	3.2 (1.6)	
Median	3.0	3.0	3.0	3.0	
**I would prefer to use message-based care with video-conferencing rather than message-based care without video-conferencing to manage my mental health**.					**<0.01** ^**2**^
*N*	284	401	300	985	
Mean (SD)	4.1 (1.5)	3.7 (1.5)	3.7 (1.5)	3.8 (1.5)	
Median	5.0	4.0	4.0	4.0	
**Telehealth usage**					
**Tele-health can play an important role in managing my mental health**.					**<0.01** ^**2**^
*N*	290	406	302	998	
Mean (SD)	4.7 (1.1)	4.7 (1.1)	4.5 (1.1)	4.6 (1.1)	
Median	5.0	5.0	5.0	5.0	
**I would use tele-health to manage my mental health**.					**<0.01** ^**2**^
*N*	288	405	302	995	
Mean (SD)	4.5 (1.3)	4.5 (1.2)	4.2 (1.3)	4.4 (1.3)	
Median	5.0	5.0	4.0	5.0	
**Tele-health can be an effective intervention for managing mental health conditions**.					**<0.01** ^**2**^
*N*	285	404	299	988	
Mean (SD)	4.7 (1.0)	4.6 (1.1)	4.3 (1.2)	4.6 (1.1)	
Median	5.0	5.0	5.0	5.0	
**If science found that tele-health is effective for managing mental health conditions, I would use it**.					**<0.01** ^**2**^
*N*	284	405	301	990	
Mean (SD)	4.7 (1.2)	4.7 (1.2)	4.3 (1.3)	4.6 (1.2)	
Median	5.0	5.0	5.0	5.0	

1 *Chi-Square p-value*;

2 *Kruskal-Wallis p-value. **Bold** indicates P-value < 0.05 and less than Benjamini-Hochberg critical value, considered to be statistically significant*.

a *Among those who have considered using an app for their mental health. Note: scale range 1–6; higher score reflect agreement*.

##### Mental Health Apps

When asked *Have you considered using an app for your mental health?* one in three participants (334/988, 33.8%) said they had considered using an app for this purpose. Compared to younger and middle-aged adults, significantly fewer older adults considered an app for their mental health ($\chi_{2}^{2}$ = 91.6, *p* < 0.01).

Among those who have considered using an app for their mental health, about a third (115/334, 34.4%) indicated that they have downloaded a mental health app, with no differences by age found.

##### Message-Based Care, With and Without Video Conferencing

Although older adults were similar to younger and middle-aged adults in their general view of mental health apps, they were less likely to rank them (1) as playing an important role in managing mental health ($\chi_{2}^{2}$ = 11.1, *p* < 0.01), (2) to manage mental health ($\chi_{2}^{2}$ = 15.7, *p* < 0.01), (3) as an effective intervention for managing mental health conditions ($\chi_{2}^{2}$ = 14.6, *p* < 0.01), and (4) were less willing to use these tools ($\chi_{2}^{2}$ = 25.2, *p* < 0.01).

When individuals were asked their preference between message-based care with or without video-conferencing, all age groups ranked supplementing message-based care with video-conferencing more favorably than message-based care without video-conferencing.

##### Tele-Mental Health

Older adults were less likely to positively rank telehealth care than younger and middle age adults in (1) playing an important role in managing mental health ($\chi_{2}^{2}$ = 11.8, *p* < 0.01), (2) used to manage mental health ($\chi_{2}^{2}$ = 15.6, *p* < 0.01), (3) effective intervention for managing mental health conditions ($\chi_{2}^{2}$ = 13.7, *p* < 0.01), and (4) willingness to use ($\chi_{2}^{2}$ = 17.9, *p* < 0.01). However, all age groups viewed this mode of care favorably.

#### Preferences for In-person, Tele-Mental Health, Message-Based Care and Mental Health Apps

Table 3 displays treatment preferences across age groups.

**Table 3:** Treatment preference stratified by age (categorized).

	**Age Categorized**				
	**Younger Adults (18-34 years) (*n* = 290)**	**Middle Aged Adults (35-59 years) (*n* = 406)**	**Older Adults (60+ years) (*n* = 302)**	**Total (*N* = 998)**	***P*-value**
**Have you ever received help in the past for stress or mental health issues? The help may have been psychotherapy, counseling, and/or medication for depression, anxiety, stress management or other mental health issue**					**<0.01** ^**1**^
No	93 (32.4%)	104 (25.7%)	61 (20.3%)	258 (26.0%)	
Yes	194 (67.6%)	301 (74.3%)	240 (79.7%)	735 (74.0%)	
Missing	3	1	1	5	
**Are you currently receiving help (psychotherapy, counseling, medication) for stress or mental health issue**					0.85^1^
No	184 (63.9%)	257 (63.5%)	197 (65.4%)	638 (64.2%)	
Yes	104 (36.1%)	148 (36.5%)	104 (34.6%)	356 (35.8%)	
Missing	2	1	1	4	
**Have you ever experienced a mental health condition, such as depression, anxiety, or psychosis?**					0.17^1^
No	39 (13.8%)	73 (18.3%)	58 (19.3%)	170 (17.3%)	
Yes	244 (86.2%)	327 (81.8%)	242 (80.7%)	813 (82.7%)	
Missing	7	6	2	15	
**Suppose all types of counseling described above are equally effective, which would you be most likely to choose**					**<0.01** ^**1**^
One-to-one in-person therapy	169 (59.1%)	183 (45.9%)	168 (56.6%)	520 (53.0%)	
Tele-health	54 (18.9%)	123 (31.6%)	75 (25.3%)	255 (26.0%)	
Message-based Care	39 (13.6%)	44 (11.0%)	38 (12.8%)	121 (12.3%)	
Mobile Mental Health App	24 (8.4%)	46 (11.5%)	16 (5.4%)	86 (8.8%)	
Missing	4	7	5	16	
**Do you think you would have any concerns about these options?**					
Message-based Care	115 (39.7%)	180 (44.3%)	141 (46.7%)	436 (43.7%)	0.21^1^
Mobile Mental Health Apps	90 (31.0%)	155 (38.2%)	138 (45.7%)	383 (38.4%)	**<0.01** ^**1**^
Tele-health	93 (32.1%)	105 (25.9%)	62 (20.5%)	260 (26.1%)	**<0.01** ^**1**^
Prefer not to answer	93 (32.1%)	105 (25.9%)	62 (20.5%)	260 (26.1%)	**<0.01** ^**1**^
One-to-one in-person therapy / counseling from a licensed clinician	34 (11.7%)	48 (11.8%)	41 (13.6%)	123 (12.3%)	0.73^1^

1 *Chi-Square p-value; **Bold** indicates P-value < 0.05 and less than Benjamini-Hochberg critical value, considered to be statistically significant*.

Participant's preferences for the mode of treatment delivery differed significantly by age ($\chi_{6}^{2}$ = 25.4, *p* < 0.01). While all three group's leading choice of treatment was in-person therapy, younger and older adults were more likely to rank this as a leading preference compared to middle aged adults, who were more likely to prefer tele-health and mobile mental health apps.

##### Concerns Over Mental Health Apps, Message-Based Care and Tele-Mental Health

All age groups indicated the greatest concern with message-based care and mobile mental health apps. Older adults in particular had greater concerns for mobile mental health apps ($\chi_{2}^{2}$ =13.5, *p* < 0.01) and telehealth ($\chi_{2}^{2}$ = 13.9, *p* < 0.01), than younger and middle age adults.

##### The Role of Technology in Mental Health Care

Participants were asked to provide open-ended responses to the question “*In your own words, what role should technology play in your mental health care?”*

Nearly all (*N* = 954) participants responded to this question. Our qualitative analysis of responses found that a majority of the total sample (56%) felt that technology should play a complementary role to traditional mental health care, with 19.8% recommending technology play a major role in mental health care. This finding was shared across age groups, with one exception: older adults were more likely to indicate that technology should play no role in mental health care (10.8%), whereas only 4.9% of younger adults and 7.5% of middle-aged adults felt technology should play no role in mental health care. See Table 4.

**Table 4:** Role size and themes mentioned in qualitative responses.

**Role Size**	**YA 8–34**		**MAA 35–59**		**OA 60+**		**Whole group**	
	***N***	**%**	***N***	**%**	***N***	**%**	***N***	**%**
None	14	4.9	29	7.5	31	10.8	74	7.8
Small or limited scope	43	15.2	54	14.0	60	21.0	157	16.5
General or Complementary	159	56.2	224	58.2	151	52.8	534	56.0
Large	67	23.7	78	20.3	44	15.4	189	19.8
**Themes mentioned**								
Paired with a Provider	74	25.5	118	29.1	100	33.1	292	29.3
For Comm and Admin	45	15.5	50	12.3	57	18.9	153	15.2
Monitoring	25	8.6	42	10.3	27	8.9	94	9.3
Access to care	101	32.5	130	41.8	80	25.7	311	31.1
Specific recommendation	70	24.1	110	27.1	79	26.2	259	26.0

Five major themes emerged regarding the specific role that technology should play in mental health care: paired with a mental health professional, for communication purposes, symptom monitoring, improving access to care, and specific recommendations for technology in mental health. Nearly a third of respondents (29.3%) indicated that technology should be paired with a mental health professional, with 33.1% of older adults endorsing this, compared to 25.5% of younger adults and 29.1% of middle-aged adults endorsing this theme. One older adult participant responded, “I think technology can be very useful with my mental health care, however, I still want that human interaction. I feel the information gathered would be a great tool for a health professional in aiding in treatment.” Similarly, technology as a means of addressing barriers to access (e.g., expanded hours, ready documentation about illness, increased quality of care) was endorsed by 31.1% of the sample, although fewer older adults (25.7%) endorsed this theme than the other age groups (see Table 4). One participant wrote, “Make it easier to access care immediately or as needed. Remove barriers or reduce such as cost, time, distance.” Approximately 15% of the total sample felt technology should be used for communication and administrative purposes, with more older adults endorsing this theme (18.9%) than other age groups. One older adult responded, “For me, scheduling appointments, alerting for appointments, initial intake data to get set up with a counselor or clinician.” Symptom monitoring was only endorsed in 9.3% of the sample, with minimal differences between age groups. A participant responded, “I think technology can serve as a watchdog and an alert for changes in mental health or like a warning signal if difficulties arise that I may not be able to handle.” See Table 4.

Finally, 26% of the sample who answered this question provided detailed recommendations for technology use. These recommendations were to use technology to promote and reinforce healthy behaviors, utilize specific applications to help mental health, and for disorders that would specifically benefit from technology, such anxiety. One participant suggested, “I am impressed by Woebot and hope that with AI [artificial intelligence] even better mental health apps become available because I would use them every day.”

## Discussion

To our knowledge, this is the first study to directly ask potential end-users of digital mental health tools the role they feel technology should serve in mental health care. This is also one of the first studies to intentionally include the voice of older adult populations in the discussion of such technology in their mental health care, and to compare older adult responses to those of younger adults. A major finding of this study is that with a few exceptions, younger, middle-age and older adults have similar perspectives on the use of digital mental health tools, and in some circumstances, older and younger adults are more aligned in their view of such tools than would be expected, given the vastly different presence of technology in their lives. Overall, all generational groups felt digital mental health tools should serve a supportive role to traditionally delivered mental health care, with technology being used primarily for extended access to clinicians, a means of communication, and, to a lesser degree, symptom monitoring. There were a few generational differences, with older adults rating message-based care and telehealth as less acceptable than their younger counterparts, and older adults were less likely to consider using an app to manage their mental health care than the younger and middle-aged respondents. These results are consistent with the overall lower use of technology by older adults (37–40). We found that across all groups the leading preference for mental health treatment was in-person therapy which is consistent with prior research on DMH preferences of adults under 68 (41), although middle-aged adults were more likely to endorse using such tools compared to younger and older adults. In sum, our results suggest that few people in need turn to digital mental health tools because the tools available do not align with how people feel these tools should be used in mental health care.

### Generational Lifestyles, Preferences, and Needs

Variation in responses among the three age groups in this study may be attributable to generational differences in life circumstances and work/social demands. Middle-aged adults are more likely to be faced with high productivity demands from work and home, given this is a stage in life where people are managing both young and old family members with growing families while juggling the demands of a career, financial pressures, and increasing health problems (30). This age group's greater acceptability of digital mental health-based solutions may reflect their need for efficient care that allows them to prioritize other life matters. Of particular interest is the finding that the majority (93.7%) of younger adults endorsed a preference for in-person therapy over technology-based care, more so than both middle aged and older adults. Although younger adult's technology use is high, our study suggests that younger adults see technology serving a specific role in their lives that does not include managing mental health issues.

For older adults, their barriers to technology, little understanding of DMH, and low-tech skills may explain part of why acceptability of DMH is lower than middle-aged adults and they prefer one-to-one therapy (19, 39). Additionally, previous research shows privacy concerns are significant hinderances to using technology (39, 42).

### Suggestions for Successful Implementation of Digital Mental Health Across the Generations

Our results suggest that digital mental health interventions should be part of a multimodal package of care that combines aspects of in-person treatment with technology (43). Accounting for patient choice in their mental health care is associated with positive outcomes in therapeutic interventions and lower drop-out rates (44). Some older adults raised concerns about the “automation” of decisions and courses of action being implemented in care when utilizing digital mental health tools, thus inclusion of patient choice and autonomy is critical (18) Participants in our study saw successful implementation of digital mental health as augmentative to their current treatment. These insights reinforce the importance of digital mental health as one component of a mental health intervention, and to the importance of vetting digital mental health tools with intended user groups, in order to create tools that will have the greatest likelihood of use (10).

## Limitations

This study has a few limitations worth mentioning. First, while we stratified recruitment to reflect US census reports of racial demographic breakdown, ultimately, sample is not entirely representative of the demographic composition for the US population. Underrepresented racial and ethnic groups in this survey include African American, Asian, and American Indian or Alaska Native or Indigenous. The older adult sample was particularly over-representative of the White racial group relative to the US census. Additionally, these findings cannot be generalized to non-English speaking individuals. Second, our sample was recruited from an online research community, which is naturally more experienced and inclined to participate in online, digital research. Thus, the perspectives reported here are from populations who may be more comfortable using technology and may also be more aware of the limitations and problems related to digital technology. Thirdly, the participant's experience with mental health services and previous treatment exceeds that of the general population which means our results on preference for treatment are not entirely representative (45). Finally, in many respects, participants were asked to reflect on mental health tools they may not have encountered before (e.g., message-based care) and were thus likely providing initial reactions to the role such tools should play in mental health care. Future research should conduct more in-depth user testing of existing tools to determine if the perspectives reported here are still valid after exposure to using these tools.

## Conclusion

Digital mental health interventions provide both older adults and younger generations fruitful ways to prevent, assess, treat, and manage mental health conditions. In this study we ascertained important generational views on how adults view technology in conjunction with their mental healthcare. While most adults were supportive of effective digital mental health technologies, our results indicate that there is no “one-size-fits-all” for mental health care and older adults were less accepting of digital mental health than other generations. Adults see value in these technologies through administrative functions such as scheduling, treatment monitoring and the ability to message their provider. The unique preferences and lifestyles of different generations demonstrate the need for variance in mental health care offerings. As the older adult population increases, researchers and systems of care have the responsibility to address patient preferences for care and recognize the barriers and challenges to the use of digital mental health tools.

## Data Availability Statement

The raw data supporting the conclusions of this article will be made available by the authors, without undue reservation.

## Ethics Statement

The studies involving human participants were reviewed and approved by University of Washington's Institutional Review Board. The patients/participants provided their written informed consent to participate in this study.

## Author Contributions

PA and BM contributed to conception and design of the study. CR, NS, and TG organized the database. MJ performed the statistical analysis. NS, MW, and TG performed the qualitative coding and analysis. MW, MJ, NS, PA, and CR wrote sections of the manuscript. All authors contributed to manuscript revision, read, and approved the submitted version.

## Funding

This project was funded by the University of Washington Department of Psychiatry and Behavioral Sciences. This study was supported in part by the Institute of Translational Health Science (ITHS) grant support (UL1 TR002319 NCATS/NIH).

## Conflict of Interest

The authors declare that the research was conducted in the absence of any commercial or financial relationships that could be construed as a potential conflict of interest.

## Publisher's Note

All claims expressed in this article are solely those of the authors and do not necessarily represent those of their affiliated organizations, or those of the publisher, the editors and the reviewers. Any product that may be evaluated in this article, or claim that may be made by its manufacturer, is not guaranteed or endorsed by the publisher.
