# Supplementary material for: Generational Perspectives on Technology's Role in Mental Health Care: A Survey of Adults With Lived Mental Health Experience
Source: Front Digit Health. 2022 Feb 10;4:840169. doi: 10.3389/fdgth.2022.840169 (PMC8868823; doi:10.3389/fdgth.2022.840169)
Supplement: Supplementary file 1 [file Data_Sheet_1.docx]

Supplementary Material

# Survey

Older Adult Use of Technology for Mental Health Survey

Introduction: Thank you for volunteering to complete our survey. We are interested in learning more about how people use technology and their thoughts and experiences with using this technology to help with improving memory, mood and well-being. Let’s start first by getting to know you.

**Section 1. Demographics**

1. How old are you?
   1. 18-150 *(150 to allow for no upper limit)*
2. What is your zip code?
3. What is your employment level?
   1. Unemployed
   2. Unpaid work at home (e.g., primary unpaid caregiver of family member)
   3. Unpaid work out of the home (e.g., volunteerism)
   4. Part time paid work outside the house
   5. Part time paid work at home
   6. Full time paid work outside the house
   7. Full time paid work at home
4. How many people, other than you, live in your house?
   1. 0
   2. 1
   3. 2
   4. 3
   5. 4
   6. 5+
5. The way that we think and ask about demographics, particularly, race, ethnicity, gender and sexual identity is evolving as more underrepresented voice are being heard about identification. Do you feel that you identify as an underrepresented population based on your skin color, heritage, socio-economic status, gender-identity, sexual orientation, or other aspects of your identity?
   1. Yes
   2. No
   3. Not sure
6. What is your race (check all that apply)?
   1. American Indian or Alaska Native or Indigenous
   2. Asian
   3. Black or African American
   4. Hispanic/Latinx
   5. Native Hawaiian/Pacific Islander
   6. Middle Eastern or North African
   7. White
7. What is your ethnicity?
   1. Hispanic/Latinx
   2. Non-Hispanic/Latin
8. What is your gender identity?
   1. Female
   2. Male
   3. Transgender, Nonbinary, or gender-nonconforming
9. Thinking about your money situation, would you say you
   1. Can’t make ends meet
   2. Have just enough to get by
   3. Are comfortable

You finished the first section of the survey, way to go!

<iframe src="https://giphy.com/embed/ely3apij36BJhoZ234" width="480" height="480" frameBorder="0" class="giphy-embed" allowFullScreen></iframe><p><a href="https://giphy.com/gifs/good-job-congratulations-otter-ely3apij36BJhoZ234">via GIPHY</a></p>

**SECTION 2: Global Health**

1. In general, would you say your health is
   1. Excellent
   2. Very good
   3. Good
   4. Fair
   5. Poor
2. In general, would you say your quality of life is
   1. Excellent
   2. Very good
   3. Good
   4. Fair
   5. Poor
3. In general, how would you rate your physical health?
   1. Excellent
   2. Very good
   3. Good
   4. Fair
   5. Poor
4. In general, how would you rate your mental health, including your mood and your ability to think?
   1. Excellent
   2. Very good
   3. Good
   4. Fair
   5. Poor
5. In general, how would you rate your satisfaction with your social activities and relationships?
   1. Excellent
   2. Very good
   3. Good
   4. Fair
   5. Poor
6. In general, please rate how well you carry out your social activities and roles. (This includes activities at home, at work, and in your community, and responsibilities as a parent, child, spouse, employee, friend, etc.)
   1. Excellent
   2. Very good
   3. Good
   4. Fair
   5. Poor
7. To what extent are you able to carry out your everyday physical activities such as walking, climbing stairs, carrying groceries, or moving a chair?
   1. Excellent
   2. Very good
   3. Good
   4. Fair
   5. Poor
8. In the past 7 days, how often have you been bothered by emotional problems such as feeling anxious, depressed, lonely, or irritable?
   1. Never
   2. Rarely
   3. Sometimes
   4. Often
   5. Always
9. In the past 7 days, how would you rate your fatigue on average?
   1. None
   2. Mild
   3. Moderate
   4. Severe
   5. Very Severe
10. In the past 7 days, how would you rate your pain on average?
    1. 0 (no pain)
    2. 1
    3. 2
    4. 3
    5. 4
    6. 5
    7. 6
    8. 7
    9. 8
    10. 9
    11. 10 (worst pain imaginable)

**Now we are going to ask you some questions about how you have been feeling over the past two weeks.**

1. How often in the past two weeks have you been bothered by little interest or pleasure in doing things?
   1. Not at all
   2. Several days
   3. More than half the days
   4. Nearly every day
2. How often in the past two weeks have you been bothered by feeling down, depressed, or hopeless?
   1. Not at all
   2. Several days
   3. More than half the days
   4. Nearly every day
3. How often in the past two weeks have you been bothered by trouble falling or staying asleep, or sleeping too much?
   1. Not at all
   2. Several days
   3. More than half the days
   4. Nearly every day
4. How often in the past two weeks have you been bothered by feeling tired or having little interest?
   1. Not at all
   2. Several days
   3. More than half the days
   4. Nearly every day
5. How often in the past two weeks have you been bothered by poor appetite or overeating?
   1. Not at all
   2. Several days
   3. More than half the days
   4. Nearly every day
6. How often in the past two weeks have you been bothered by feeling bad about yourself – or that you are a failure or have let yourself or your family down?
   1. Not at all
   2. Several days
   3. More than half the days
   4. Nearly every day
7. How often in the past two weeks have you been bothered by trouble concentrating on things, such as reading the newspaper or watching television?
   1. Not at all
   2. Several days
   3. More than half the days
   4. Nearly every day
8. How often in the past two weeks have you been bothered by moving or speaking so slowly that other people could have noticed? Or so fidgety or restless that you have been moving a lot more than usual?
   1. Not at all
   2. Several days
   3. More than half the days
   4. Nearly every day
9. How often in the past two weeks have you been bothered by thoughts that you would be better off dead, or thoughts of hurting yourself in some way?
   1. Not at all
   2. Several days
      1. If they select ‘Several Days’, show the following text: “Your response to this question indicates you may be at risk for harming yourself or someone else. Are you in crisis? Please call 911 or the National Suicide Prevention Hotline at 1-800-273-TALK or go immediately to the nearest emergency room.”
   3. More than half the days
      1. If they select ‘More than half the days’, show the following text: “Your response to this question indicates you may be at risk for harming yourself or someone else. Are you in crisis? Please call 911 or the National Suicide Prevention Hotline at 1-800-273-TALK or go immediately to the nearest emergency room.”
   4. Nearly every day
      1. If they select ‘Nearly every day’, show the following text: “Your response to this question indicates you may be at risk for harming yourself or someone else. Are you in crisis? Please call 911 or the National Suicide Prevention Hotline at 1-800-273-TALK or go immediately to the nearest emergency room.”

**GAD-7**

1. How often in the past two weeks have you been bothered by feeling nervous, anxious, or on edge?
   1. Not at all
   2. Several days
   3. More than half the days
   4. Nearly every day
2. How often in the past two weeks have you been bothered by not being able to stop or control worrying?
   1. Not at all
   2. Several days
   3. More than half the days
   4. Nearly every day
3. How often in the past two weeks have you been bothered by worrying too much about different things?
   1. Not at all
   2. Several days
   3. More than half the days
   4. Nearly every day
4. How often in the past two weeks have you been bothered by trouble relaxing?
   1. Not at all
   2. Several days
   3. More than half the days
   4. Nearly every day
5. How often in the past two weeks have you been bothered by being so restless that it's hard to sit still?
   1. Not at all
   2. Several days
   3. More than half the days
   4. Nearly every day
6. How often in the past two weeks have you been bothered by becoming easily annoyed or irritable?
   1. Not at all
   2. Several days
   3. More than half the days
   4. Nearly every day
7. How often in the past two weeks have you been bothered by feeling afraid as if something awful might happen?
   1. Not at all
   2. Several days
   3. More than half the days
   4. Nearly every day

Thank you for your efforts so far!

<iframe src="https://giphy.com/embed/fUA19fi3gRyxmpLb8I" width="480" height="360" frameBorder="0" class="giphy-embed" allowFullScreen></iframe><p><a href="https://giphy.com/gifs/star-health-mat-voyce-fUA19fi3gRyxmpLb8I">via GIPHY</a></p>

**SECTION 3: Use of technology**

1. Do you have access to a computer or laptop? Yes/No [If NO, go to question 37]
   1. Is this computer/laptop at home? Y/N
   2. Do you share this computer/laptop with other people/ Y/N
      1. If yes: how many other people use this computer/laptop?
   3. How often do you use this computer/laptop?
      1. At least once everyday
      2. Every other day
      3. Less than three times a week
      4. Once a week
      5. Once a month
      6. Hardly ever
      7. Never
   4. What do you use your computer or laptop for? (select all that apply)
      1. Searching for information
      2. Writing email messages
      3. Sending text messages
      4. Watching movies or videos
      5. Banking or other financial services
      6. Gaming
      7. Reading books
      8. Social media
      9. Browsing the internet
      10. Contact family through video conferencing (like Zoom)
      11. Contact friends through video conferencing
      12. Contact my doctor through video conferencing
      13. Other (write in)
2. Do you have access to a smart phone? Y/N [If NO, go to Question 38]
   1. Is it your personal phone? By personal phone, we mean a phone that is managed just by you and not your employer, school or other organization. Y/N
   2. Does anyone else use this phone on a regular basis (family of friends)? Y/N
      1. If yes, how many other people use the phone? [0, 1, 2, 3, 4, 5+]
      2. If yes, how often do you hand someone this phone on a given day? [Never, once, twice, three times, four times, five or more times]
   3. How often do you use your personal phone?
      1. At least once everyday
      2. Every other day
      3. Less than three times a week
      4. Once a week
      5. Once a month
      6. Hardly ever
      7. Never
   4. What do you use your phone for? (check all that apply)
      1. Searching for information
      2. Writing email messages
      3. Sending text messages
      4. Watching movies or videos
      5. Banking or other financial services
      6. Gaming
      7. Reading books
      8. Social media
      9. Browsing the internet
      10. Contact family through a phone call
      11. Contact friends through a phone call
      12. Contact my doctor through a phone call
      13. Contact family through video conferencing (like Zoom)
      14. Contact friends through video conferencing
      15. Contact my doctor through video conferencing
      16. Other (write in)
3. Do you have access to a smart tablet (like an iPad or Kindle Fire)? Y/N [If NO, go to 39]
   1. If yes: Is this your personal tablet? By personal tablet, we mean a tablet that is managed just by you and not your employer, school or other organization. Y/N
   2. Does anyone else use this tablet on a regular basis (family or friends)? Y/N
      1. If yes, how many other people use the phone? [0, 1, 2, 3, 4, 5+]
      2. If yes, how often do you hand someone this phone on a given day? [Never, once, twice, three times, four times, five or more times]
   3. How often do you use your smart tablet?
      1. At least once everyday
      2. Every other day
      3. Less than three times a week
      4. Once a week
      5. Once a month
      6. Hardly ever
      7. Never
   4. What do you use your tablet for? (check all that apply)
      1. Searching for information
      2. Writing email messages
      3. Sending text messages
      4. Watching movies or videos
      5. Banking or other financial services
      6. Gaming
      7. Reading books
      8. Social media
      9. Browsing the internet
      10. Contact family through video conferencing (like Zoom)
      11. Contact friends through video conferencing
      12. Contact my doctor through video conferencing
      13. Other (write in)
4. Do you use video chat technology (e.g., FaceTime, Duo, Google Hangout, video conferencing)?
   1. Yes
   2. No
      1. Why not? (Select all that apply)
         1. I do not like using video for communicating.
         2. I do not have a device with a camera.
         3. I do not have the privacy necessary for video chatting.
         4. Other (*open field*)

- Many people experience challenges using technology, even when they are comfortable using technology. It is not unusual for changes in vision, motor speed and dexterity to influence the usability of modern technology. For each statement below, please indicate the frequency of your experiences with technology in general and for specific tools:

1. I find it hard to read information on **computers/laptops**.
   1. Never
   2. Rarely
   3. Occasionally/Sometimes
   4. A lot
   5. Always
2. I find it hard to read information on **smartphones**.
   1. Never
   2. Rarely
   3. Occasionally/Sometimes
   4. A lot
   5. Always
3. I find it hard to read information on **tablets**.
   1. Never
   2. Rarely
   3. Occasionally/Sometimes
   4. A lot
   5. Always
4. I find it hard to type messages (email, text) on **computers/laptops**.
   1. Never
   2. Rarely
      1. I find it hard to type messages on computers/laptops because I experience:
         1. Pain in my body
         2. I keep making mistakes when typing
         3. Other [*open field*]
   3. Occasionally/Sometimes
      1. I find it hard to type messages on computers/laptops because I experience:
         1. Pain in my body
         2. I keep making mistakes when typing
         3. Other:
   4. A lot
      1. I find it hard to type messages on computers/laptops because I experience:
         1. Pain in my body
         2. I keep making mistakes when typing
         3. Other:
   5. Always
      1. I find it hard to type messages on computers/laptops because I experience:
         1. Pain in my body
         2. I keep making mistakes when typing
         3. Other:
   6. N/A – I do not use computers to type
5. I find it hard to type on **smartphones**.
   1. Never
   2. Rarely
      1. I find it hard to type messages on smartphones because I experience:
         1. Pain in my body
         2. I keep making mistakes when typing
         3. Other:
   3. Occasionally/Sometimes
      1. I find it hard to type messages on smartphones because I experience:
         1. Pain in my body
         2. I keep making mistakes when typing
         3. Other:
   4. A lot
      1. I find it hard to type messages on smartphones because I experience:
         1. Pain in my body
         2. I keep making mistakes when typing
         3. Other:
   5. Always
      1. I find it hard to type messages on smartphones because I experience:
         1. Pain in my body
         2. I keep making mistakes when typing
         3. Other:
   6. N/A – I do not use smartphones to type
6. I find it hard to type on **tablets**.
   1. Never
   2. Rarely
      1. I find it hard to type messages on tablets because I experience:
         1. Pain in my body
         2. I keep making mistakes when typing
         3. Other:
   3. Occasionally/Sometimes
      1. I find it hard to type messages on tablets because I experience:
         1. Pain in my body
         2. I keep making mistakes when typing
         3. Other:
   4. A lot
      1. I find it hard to type messages on tablets because I experience:
         1. Pain in my body
         2. I keep making mistakes when typing
         3. Other:
   5. Always
      1. I find it hard to type messages on tablets because I experience:
         1. Pain in my body
         2. I keep making mistakes when typing
         3. Other:
   6. N/A – I do not use tablets to type
7. I find **computers** too confusing to use.
   1. Never
   2. Rarely
      1. I find computers too confusing to use because the equipment (for example, the mouse, keyboard, or screen) is confusing.
      2. I find computers too confusing to use because computer programs, software, or applications are confusing.
   3. Occasionally/Sometimes
      1. I find computers too confusing to use because the equipment (for example, the mouse, keyboard, or screen) is confusing.
      2. I find computers too confusing to use because computer programs, software, or applications are confusing.
   4. A lot
      1. I find computers too confusing to use because the equipment (for example, the mouse, keyboard, or screen) is confusing.
      2. I find computers too confusing to use because computer programs, software, or applications are confusing.
   5. Always
      1. I find computers too confusing to use because the equipment (for example, the mouse, keyboard, or screen) is confusing.
      2. I find computers too confusing to use because computer programs, software, or applications are confusing.
8. I find **smartphones** too confusing to use.
   1. Never
   2. Rarely
      1. I find smartphones too confusing to use because the equipment (for example, the phone, charger, or headphones) is confusing.
      2. I find smartphones too confusing to use because programs, software, or applications are confusing.
   3. Occasionally/Sometimes
      1. I find smartphones too confusing to use because the equipment (for example, the phone, charger, or headphones) is confusing.
      2. I find smartphones too confusing to use because programs, software, or applications are confusing.
   4. A lot
      1. I find smartphones too confusing to use because the equipment (for example, the phone, charger, or headphones) is confusing.
      2. I find smartphones too confusing to use because programs, software, or applications are confusing.
   5. Always
      1. I find smartphones too confusing to use because the equipment (for example, the phone, charger, or headphones) is confusing.
      2. I find smartphones too confusing to use because programs, software, or applications are confusing.
9. I find **tablets** too confusing to use.
   1. Never
   2. Rarely
      1. I find tablets too confusing to use because the equipment (for example, the tablet or charger) is confusing.
      2. I find tablets too confusing to use because programs, software, or applications are confusing.
   3. Occasionally/Sometimes
      1. I find tablets too confusing to use because the equipment (for example, the tablet or charger) is confusing.
      2. I find tablets too confusing to use because programs, software, or applications are confusing.
   4. A lot
      1. I find tablets too confusing to use because the equipment (for example, the tablet or charger) is confusing.
      2. I find tablets too confusing to use because programs, software, or applications are confusing.
   5. Always
      1. I find tablets too confusing to use because the equipment (for example, the tablet, charger, or headphones) is confusing.
      2. I find tablets too confusing to use because programs, software, or applications are confusing.
10. Please select “A lot” for this item.
    1. Never
    2. Rarely
    3. Occasionally/Sometimes
    4. A lot
    5. Always
11. It is too hard to manage new updates to **computers**.
    1. Never
    2. Rarely
    3. Occasionally/Sometimes
    4. A lot
    5. Always
12. It is too hard to manage new updates to **smartphones**.
    1. Never
    2. Rarely
    3. Occasionally/Sometimes
    4. A lot
    5. Always
13. It is too hard to manage new updates to **tablets**.
    1. Never
    2. Rarely
    3. Occasionally/Sometimes
    4. A lot
    5. Always

Keep up the good work!

<iframe src="https://giphy.com/embed/sx3LonKdVZG1i" width="480" height="375" frameBorder="0" class="giphy-embed" allowFullScreen></iframe><p><a href="https://giphy.com/gifs/reaction-vintage-classic-sx3LonKdVZG1i">via GIPHY</a></p>

**SECTION 4: PRIVACY AND SECURITY. Some people prefer not to use technology because of concerns about privacy and security concerns**.

1. In general, how concerned are you about security on the Internet (e.g. people reading your email, finding out what websites you visit, etc.)? Keep in mind that "security" can mean **privacy, confidentiality, and/or proof of identity** for you or for someone else.
   1. Not at all concerned
   2. A little concerned
   3. Somewhat concerned
   4. Very concerned
   5. I know I should be concerned, but I’m not
2. How concerned are you about security in relation to making purchases or banking over the Internet?
   1. Not at all concerned
   2. A little concerned
   3. Somewhat concerned
   4. Very concerned
   5. I know I should be concerned, but I’m not
3. How concerned are you about security in relation to communicating with your health provider over the Internet?
   1. Not at all concerned
   2. A little concerned
   3. Somewhat concerned
   4. Very concerned
   5. I know I should be concerned, but I’m not

1. How concerned are you about security in relation to talking to your health care provider over Text Messaging?
   1. Not at all concerned
   2. A little concerned
   3. Somewhat concerned
   4. Very concerned
   5. I know I should be concerned, but I’m not
2. How concerned are you about security in relation to talking to your health care provider over email?
   1. Not at all concerned
   2. A little concerned
   3. Somewhat concerned
   4. Very concerned
   5. I know I should be concerned, but I’m not
3. How concerned are you about security in relation to talking to your health care provider over secure video conferencing (Zoom, Skype, etc.)?
   1. Not at all concerned
   2. A little concerned
   3. Somewhat concerned
   4. Very concerned
   5. I know I should be concerned, but I’m not
4. How concerned are you about security in relation looking for health or mental health information over the Internet?
   1. Not at all concerned
   2. A little concerned
   3. Somewhat concerned
   4. Very concerned
   5. I know I should be concerned, but I’m not
5. Could technology providers and/or app creators provide any information that would allow you relieve your concerns about privacy and security?
   1. Yes
      1. Please describe what information would be necessary to relieve your concerns about privacy and security. [*open text box]*
      2. How would you prefer to receive this information?
         1. Acceptance of terms and conditions prior to use
         2. Informational video prior to use
         3. Interactive communication (e.g., text messaging, responding to quizzes) prior to use
         4. Frequent updates on privacy and security efforts taken by the company
         5. Other [*open text box]*
   2. No
   3. I’m not sure
      1. Please consider providing more information about your response. [*open text box*]

You are about a third of the way through the survey, good work!

<iframe src="https://giphy.com/embed/26tnaxoeNOaGkT88E" width="480" height="270" frameBorder="0" class="giphy-embed" allowFullScreen></iframe><p><a href="https://giphy.com/gifs/serena-williams-thumbs-up-26tnaxoeNOaGkT88E">via GIPHY</a></p>

**SECTION #5: USE OF APPS, SOCIAL MEDIA, INTERNET**

**In this next section we are curious about your interest in, concerns with and use of digital technology for health and mental health purposes. We will be asking you about three different types of digital health and mental health options: mobile apps, message (text) based care, and telehealth.**

**Mobile Apps:**

**Health and mental health mobile applications are smartphone or tablets apps that can help you track your symptoms, remind you to take medication or do and exercise, give you information about your condition, and provide you with motivation to help you stay on track with your health improvement plan. An app that only allows you to manage your health insurance is not what we’re looking for here! Mobile health and/or mental health apps may be entirely self-directed, or they may offer support from either licensed providers or lay persons, such as coaches or peers. Examples of health apps include Google Fit, Clue, MyFitnessPal, and FitBit. Examples of mental health apps include 7 Cups of Tea, BetterHelp, Sanvello, Talkspace, and What’s Up.**

1. How many health apps do you have on your smartphone or tablet currently?
   1. 0-150 (*to allow for no upper limit)*

Please state whether these next statements you strongly agree, agree, somewhat agree, somewhat disagree, disagree, and strongly disagree

1. Health Apps can play an important role in managing my health.
   1. Strongly agree
   2. Agree
   3. Somewhat agree
   4. Somewhat disagree
   5. Disagree
   6. Strongly disagree
2. Mental Health Apps can play and important role in managing my mental health.
   1. Strongly agree
   2. Agree
   3. Somewhat agree
   4. Somewhat disagree
   5. Disagree
   6. Strongly disagree
3. I would use a health app to manage my health.
   1. Strongly agree
   2. Agree
   3. Somewhat agree
   4. Somewhat disagree
   5. Disagree
   6. Strongly disagree
4. I would use a mental health app to manage my mental health.
   1. Strongly agree
   2. Agree
   3. Somewhat agree
   4. Somewhat disagree
   5. Disagree
   6. Strongly disagree
5. Health apps can be an effective intervention for managing health conditions.
   1. Strongly agree
   2. Agree
   3. Somewhat agree
   4. Somewhat disagree
   5. Disagree
   6. Strongly disagree
6. Mental health apps can be an effective intervention for managing mental health conditions.
   1. Strongly agree
   2. Agree
   3. Somewhat agree
   4. Somewhat disagree
   5. Disagree
   6. Strongly disagree
7. If science found that health apps were effective for managing health conditions, I would use these apps.
   1. Strongly agree
   2. Agree
   3. Somewhat agree
   4. Somewhat disagree
   5. Disagree
   6. Strongly disagree
8. If science found that mental health apps were effective for managing mental health conditions, I would use these apps.
   1. Strongly agree
   2. Agree
   3. Somewhat agree
   4. Somewhat disagree
   5. Disagree
   6. Strongly disagree

**Message (Text) Based Care. Message based care allows you the opportunity to talk with your doctor or provider about your health or mental health condition at your convenience. These tools may be accessed via computer-based programs, smartphone apps, or SMS texts. They use secure messaging, which includes the ability to leave a voice mail, e-mail, or text to your doctor about anything, from general questions to discussing private matters. Some companies offer this service in combination with video-conferencing (like Zoom or Skype, only secure).**

Please state whether these next statements you strongly agree, somewhat agree, somewhat disagree and strongly disagree

1. Message-based care can play an important role in managing my health.
   1. Strongly agree
   2. Agree
   3. Somewhat agree
   4. Somewhat disagree
   5. Disagree
   6. Strongly disagree
2. Message-based care can play and important role in managing my mental health.
   1. Strongly agree
   2. Agree
   3. Somewhat agree
   4. Somewhat disagree
   5. Disagree
   6. Strongly disagree
3. I would use message-based care to manage my health.
   1. Strongly agree
   2. Agree
   3. Somewhat agree
   4. Somewhat disagree
   5. Disagree
   6. Strongly disagree
4. I would use message-based care to manage my mental health.
   1. Strongly agree
   2. Agree
   3. Somewhat agree
   4. Somewhat disagree
   5. Disagree
   6. Strongly disagree
5. Message-based care can be an effective intervention for managing health conditions.
   1. Strongly agree
   2. Agree
   3. Somewhat agree
   4. Somewhat disagree
   5. Disagree
   6. Strongly disagree
6. Message-based care can be an effective intervention for managing mental health conditions.
   1. Strongly agree
   2. Agree
   3. Somewhat agree
   4. Somewhat disagree
   5. Disagree
   6. Strongly disagree
7. If science found that message-based care is effective for managing health conditions, I would use it.
   1. Strongly agree
   2. Agree
   3. Somewhat agree
   4. Somewhat disagree
   5. Disagree
   6. Strongly disagree
8. If science found that message-based care is effective for managing mental health conditions, I would use it.
   1. Strongly agree
   2. Agree
   3. Somewhat agree
   4. Somewhat disagree
   5. Disagree
   6. Strongly disagree
9. I believe using only message-based care, without video-conferencing, can be effective in managing my health.
   1. Strongly agree
   2. Agree
   3. Somewhat agree
   4. Somewhat disagree
   5. Disagree
   6. Strongly disagree
10. I believe using only message-based care, without video-conferencing, can be effective in managing my mental health.
    1. Strongly agree
    2. Agree
    3. Somewhat agree
    4. Somewhat disagree
    5. Disagree
    6. Strongly disagree
11. I would prefer to use message-based care (without video-conferencing) rather than video-conferencing to manage my physical health.
    1. Strongly agree
    2. Agree
    3. Somewhat agree
    4. Somewhat disagree
    5. Disagree
    6. Strongly disagree
12. I would prefer to use message-based care (without video-conferencing) rather than video-conferencing to manage my mental health.
    1. Strongly agree
    2. Agree
    3. Somewhat agree
    4. Somewhat disagree
    5. Disagree
    6. Strongly disagree
13. I would prefer to use message-based care with video-conferencing rather than message-based care without video-conferencing to manage my physical health.
    1. Strongly agree
    2. Agree
    3. Somewhat agree
    4. Somewhat disagree
    5. Disagree
    6. Strongly disagree
14. I would prefer to use message-based care with video-conferencing rather than message-based care without video-conferencing to manage my mental health.
    1. Strongly agree
    2. Agree
    3. Somewhat agree
    4. Somewhat disagree
    5. Disagree
    6. Strongly disagree

**Tele-health. Tele-mental health is the use of phones and the internet to deliver care. People can talk to their doctor or provider during scheduled visits over the phone or through video-conferencing, and can access information and tools to help with the management of a condition.**

Please state whether these next statements you strongly agree, somewhat agree, somewhat disagree and strongly disagree

1. Tele-health can play an important role in managing my health.
   1. Strongly agree
   2. Agree
   3. Somewhat agree
   4. Somewhat disagree
   5. Disagree
   6. Strongly disagree
2. Tele-health can play and important role in managing my mental health.
   1. Strongly agree
   2. Agree
   3. Somewhat agree
   4. Somewhat disagree
   5. Disagree
   6. Strongly disagree
3. I would use tele-health to manage my health.
   1. Strongly agree
   2. Agree
   3. Somewhat agree
   4. Somewhat disagree
   5. Disagree
   6. Strongly disagree
4. I would use tele-health to manage my mental health.
   1. Strongly agree
   2. Agree
   3. Somewhat agree
   4. Somewhat disagree
   5. Disagree
   6. Strongly disagree
5. Tele-health can be an effective intervention for managing health conditions.
   1. Strongly agree
   2. Agree
   3. Somewhat agree
   4. Somewhat disagree
   5. Disagree
   6. Strongly disagree
6. Tele-health can be an effective intervention for managing mental health conditions.
   1. Strongly agree
   2. Agree
   3. Somewhat agree
   4. Somewhat disagree
   5. Disagree
   6. Strongly disagree
7. If science found that tele-health is effective for managing health conditions, I would use these it.
   1. Strongly agree
   2. Agree
   3. Somewhat agree
   4. Somewhat disagree
   5. Disagree
   6. Strongly disagree
8. If science found that tele-health is effective for managing mental health conditions, I would use these it.
   1. Strongly agree
   2. Agree
   3. Somewhat agree
   4. Somewhat disagree
   5. Disagree
   6. Strongly disagree
9. Have you considered using an app for **wellness activities?** **(These include monitoring a chronic health condition, exercise, diet, or sleep. The next set of questions is how you use tech/apps for your physical health, we will ask questions about your mental health later)**
   - Yes – **GO TO QUESTION 93**
   - No - **GO TO QUESTION 100**
10. Have you downloaded a wellness app?
    - Yes – **GO TO 94**
    - No
      1. Why not? (**after response, GO TO QUESTION 100)**
         1. I didn’t think to look for an app
         2. I couldn’t find an app that was relevant to what I needed
         3. I don’t have time to use an app
         4. I don’t have money to spend on an app or on a data plan to use apps
         5. I don’t think apps would help me
         6. I prefer to work with a professional
         7. I have other ways of tracking wellness activities [open field: please list strategies]
         8. Other reason [open field]
11. Which wellness app did you download? *Please select all the apply.*
    - 1. Calm
      2. Endel
      3. Headspace
      4. Insight Timer
      5. Sleep Cycle
      6. SleepScore
      7. Ten Percent Happier
      8. Other [open field]

If you selected multiple apps in the previous question, please think about the app that you used the most when answering the following questions.

1. How satisfied are you with how the app impacted your wellness? *[Can only select one; please set this up as a scale]*
   1. Not at all satisfied
   2. Partly satisfied
   3. Satisfied
   4. More than satisfied
   5. Very satisfied
2. Did you use the app for at least a month?
   1. Yes
      1. How often did you use the app?
         1. Rarely (1-3 times in the past month)
         2. Infrequently (less than weekly)
         3. Weekly
         4. More than weekly but less than daily
         5. Daily
         6. Multiple times per day – **GO TO QUESTION 91**
   2. No
      1. When did you stop using the app?
         1. Immediately (within a day of downloading)
         2. Within the first week after downloading
         3. Within 2 weeks after downloading
         4. Between 2 and 4 weeks after downloading
      2. Why did you stop using the app?
         1. The app was not relevant to what I needed
         2. I don’t have time to use the app
         3. I don’t have money to spend on the app, app features, or on a data plan to use apps
         4. I don’t think app would help me
         5. I prefer to work with a professional
         6. I have other ways of tracking wellness activities [open field: please list strategies]
         7. Other reason [open field] – **GO TO** **QUESTION 100**
3. Are you still using the app?
   1. Yes
   2. No
      1. Why did you stop using the app?
         1. I reached my goal and no longer needed to use the app.
         2. I don’t have time to use the app.
         3. I don’t have money to spend on the app, app features, or on a data plan to use apps.
         4. I no longer think the app will help me.
         5. I prefer to work with a professional.
         6. I have other ways of tracking wellness activities [open field: please list strategies]
         7. Other reason [open field]
4. Did you stop using the app and then use it again?
   1. Yes
      1. Why?
         1. I set a new goal relevant to this app.
         2. I returned to a previous goal relevant to this app.
         3. Other [open field]
   2. No
5. Did you stop using the app and then use another wellness app?
   1. Yes
      1. Why?
         1. The previous app did not meet my needs.
         2. The previous app was too expensive.
         3. The previous app was unnecessarily complex.
         4. There was too much inconsistency in the previous app.
         5. I found the previous app very cumbersome/awkward to use.
   2. No
6. If an app could accurately track your wellness practices and provide recommendations for more effective wellness behavior, would you take the app’s advice?
   1. Yes
      1. If Yes, Why? *Select all that apply*
         1. I have confidence in the app.
         2. I have time to follow the app’s recommendations.
         3. I have the resources (e.g., money, physical space, equipment, technology) to follow the app’s recommendations.
         4. I believe that the app’s recommendations will help me meet my goal.
   2. No
      1. If no, why?
         1. I do not have confidence in the app.
         2. I do not have time to follow the app’s recommendations.
         3. I do not have the resources (e.g., money, physical space, equipment, technology) to follow the app’s recommendations.
         4. I do not believe that the app’s recommendations will help me meet my goal.
         5. I would prefer to receive recommendations on wellness from professionals (e.g., fitness instructors, coaches).
7. Have you considered using an app for your **mental health** **(e.g., depression symptoms, anxiety, risky behaviors)?**
   1. Yes – **GO TO QUESTION 102**
   2. No – **GO TO QUESTION 109**
8. Have you downloaded a mental health app?
   1. Yes – **GO TO QUESTION 102**
   2. No
      1. Why not? (**after response, GO TO QUESTION 109)**
         1. I didn’t think to look for an app
         2. I couldn’t find an app that was relevant to what I needed
         3. I don’t have time to use an app
         4. I don’t have money to spend on an app or on a data plan to use apps
         5. I don’t think apps would help me
         6. I prefer to work with a professional in person
         7. I have other ways of tracking my mental health [open field: please list strategies]
         8. Other reason [open field]
9. Which mental health app did you download? *Please select all the apply.*
   1. 7 Cups of Tea
   2. Beautiful Mood
   3. BetterHelp
   4. Happify
   5. Mood Kit
   6. PTSD Coach
   7. Sanvello
   8. Smiling Mind
   9. Talkspace
   10. What’s Up
   11. Other [open field]

If you selected multiple apps in the previous question, please think about the app that you used the most when answering the following questions.

1. How satisfied are you with how the app impacted your mental health? *[Can only select one; please set this up as a scale]*
   1. 1 – Not at all satisfied
   2. 2 – Partly satisfied
   3. 3 – Satisfied
   4. 4 – More than satisfied
   5. 5 - Very satisfied
2. Did you use the app for at least a month?
   1. Yes
      1. How often did you use the app?
         1. Rarely (1-3 times in the past month)
         2. Infrequently (less than weekly)
         3. Weekly
         4. More than weekly but less than daily
         5. Daily
         6. Multiple times per day – **GO TO QUESTION 106**
   2. No
      1. When did you stop using the app?
         1. Immediately (within a day of downloading)
         2. Within the first week after downloading
         3. Within 2 weeks after downloading
         4. Between 2 and 4 weeks after downloading
      2. Why did you stop using the app?
         1. The app was not relevant to what I needed
         2. I don’t have time to use the app
         3. I don’t have money to spend on the app, app features, or on a data plan to use apps
         4. I don’t think app would help me
         5. I prefer to work with a professional in person
         6. I have other ways of tracking my mental health [open field: please list strategies]
         7. Other reason [open field] – **GO TO** **QUESTION 109**
3. Are you still using the app?
   1. Yes
   2. No
      1. Why did you stop using the app?
         1. I reached my goal and no longer needed to use the app.
         2. I don’t have time to use the app.
         3. I don’t have money to spend on the app, app features, or on a data plan to use apps.
         4. I no longer think the app will help me.
         5. I prefer to work with a professional in person
         6. I have other ways of tracking my mental health [open field: please list strategies]
         7. Other reason [open field]
4. Did you stop using the app and then use it again?
   1. Yes
      1. Why?
         1. I set a new goal relevant to this app.
         2. I returned to a previous goal relevant to this app.
         3. Other [open field]
   2. No
5. Did you stop using the app and then use another mental health app?
   1. Yes
      1. Why?
         1. The previous app did not meet my needs.
         2. The previous app was too expensive.
         3. The previous app was unnecessarily complex.
         4. There was too much inconsistency in the previous app.
         5. I found the previous app very cumbersome/awkward to use.
   2. No
6. If an app could accurately track information about your mental health (e.g., depression, risky behaviors) and provide recommendations for steps to better your mental health, would you take the app’s advice?
   1. Yes
      1. If Yes, Why? *Select all that apply*
         1. I have confidence in the app.
         2. I have time to follow the app’s recommendations.
         3. I have the resources (e.g., money, physical space, equipment, technology) to follow the app’s recommendations.
         4. I believe that the app’s recommendations will help me meet my goal.
   2. No
      1. If no, why?
         1. I do not have confidence in the app.
         2. I do not have time to follow the app’s recommendations.
         3. I do not have the resources (e.g., money, physical space, equipment, technology) to follow the app’s recommendations.
         4. I do not believe that the app’s recommendations will help me meet my goal.
         5. I would prefer to receive recommendations on mental health from professionals (e.g., therapists, counselors, social workers, psychiatrists, psychologists).
7. Please select “Maybe” for this item.
   1. Yes
   2. No
   3. Maybe

You are in the home stretch!

**<iframe src="https://giphy.com/embed/QW5nKIoebG8y4" width="480" height="320" frameBorder="0" class="giphy-embed" allowFullScreen></iframe><p><a href="https://giphy.com/gifs/peanuts-youre-not-elected-charlie-brown-QW5nKIoebG8y4">via GIPHY</a></p>**

SECTION 6: Preferences for treatment. We now would like to ask you about your preferences for mental health treatments.

1. Have you ever received help in the past for stress or mental health issues? The help may have been psychotherapy, counseling, and/or medication for depression, anxiety, stress management or other mental health issue.
   1. Yes
   2. No
2. Are you currently receiving help (psychotherapy, counseling, medication) for stress or mental health issue?
   1. Yes
   2. No
3. Have you ever experienced a mental health condition, such as depression, anxiety, or psychosis?
   1. Yes
   2. No
4. If you decide to seek counseling in the future, please tell us what types you might be interested in:
   1. One-to-one in-person therapy (counseling from a licensed clinician
      1. Yes, I would consider using
      2. No, I would not consider
      3. Unsure
   2. Mobile Mental Health App
      1. Yes, I would consider using
      2. No, I would not consider
      3. Unsure)
   3. Message-Based Care (text-based support from a licensed clinician)
      1. Yes, I would consider using
      2. No, I would not consider
      3. Unsure)
   4. Tele-health: help from a licensed clinician through regularly scheduled secure video conferencing appointments
      1. Yes, I would consider using
      2. No, I would not consider
      3. Unsure
5. Suppose all types of counseling described above are equally effective, which would you be most likely to choose? (Select one option)
   1. One-to-one in-person therapy / counseling from a licensed clinician
   2. Mobile Mental Health App / counseling you can complete at home
   3. Message-based Care / counseling you can complete at home that also includes help as needed using an app-based or text-based support from a licensed clinician.
   4. Tele-health / counseling you can complete at home that also includes help from a licensed clinician through regularly scheduled secure video conferencing appointments.
6. Do you think you would have any concerns about these options (check all that apply)?
   1. One-to-one in-person therapy / counseling from a licensed clinician
   2. Mobile Mental Health App / counseling you can complete at home
   3. Message-based Care / counseling you can complete at home that also includes help as needed using an app-based or text-based support from a licensed clinician.
   4. Tele-health / counseling you can complete at home that also includes help from a licensed clinician through regularly scheduled secure video conferencing appointments.
   5. For those you checked, please describe any concerns. _________________________

SECTION 7: BUILD YOUR OWN MENTAL HEALTH ADVENTURE. There are many ways that technology can help you manage stress and mental wellness.

1. In your own words, what role should technology serve in mental health care?
2. The top-rated technologies for mental health are somewhat different from each other. We are interested in learning about which features you feel would help you with your emotional health. Please rank each feature below from not at all important to very important (0-15):
   1. Information or education about mental health conditions
   2. Mindfulness/meditation tools
   3. Symptom tracking (tracking sleep or mood)
   4. Medication management tools (reminders, side effect management)
   5. Brain games to improve concentration
   6. Distraction tools (drawing, puzzles, music)
   7. Tools to focus on the positive events and influences in life
   8. Link to resources, counseling, or crisis support
   9. A chatbot to help you with daily stress
   10. Access to a licensed clinician through email
   11. Access to a licensed clinician through text-messaging
   12. The ability to have regular appointments with a clinician
   13. Peer Counseling: Support from people like me who have successfully managed their mental health and have training to help people like me.
   14. Support from others like me who are like me and are currently managing their stress/mental health.
   15. Other?  [Open text field]

1. Now it’s time for you to build your own mental health experience. If you could design or create a mental health program specifically for you:
   1. What treatment and services would it include? (text box)
   2. How often would you want to interact with your provider? (text box)
   3. How long do you think it should take for you to see improvement? (text box)
   4. What kind of technology would you think would be important to include in your care, if at all? (text box)
